# Anticipating artificial intelligence in mammography screening: views of Swedish breast radiologists

**DOI:** 10.1136/bmjhci-2022-100712

**Published:** 2023-05-22

**Authors:** Charlotte Högberg, Stefan Larsson, Kristina Lång

**Affiliations:** 1 Department of Technology and Society, Lund University Faculty of Engineering, Lund, Sweden; 2 Department of Translational Medicine, Diagnostic Radiology, Lund University Faculty of Medicine, Lund, Sweden; 3 Unilabs Mammography Unit, Skåne University Hospital Lund, Malmö, Sweden

**Keywords:** Artificial intelligence, Decision Making, Computer-Assisted, Decision Support Systems, Clinical, Machine Learning

## Abstract

**Objectives:**

Artificial intelligence (AI) is increasingly tested and integrated into breast cancer screening. Still, there are unresolved issues regarding its possible ethical, social and legal impacts. Furthermore, the perspectives of different actors are lacking. This study investigates the views of breast radiologists on AI-supported mammography screening, with a focus on attitudes, perceived benefits and risks, accountability of AI use, and potential impact on the profession.

**Methods:**

We conducted an online survey of Swedish breast radiologists. As early adopter of breast cancer screening, and digital technologies, Sweden is a particularly interesting case to study. The survey had different themes, including: attitudes and responsibilities pertaining to AI, and AI’s impact on the profession. Responses were analysed using descriptive statistics and correlation analyses. Free texts and comments were analysed using an inductive approach.

**Results:**

Overall, respondents (47/105, response rate 44.8%) were highly experienced in breast imaging and had a mixed knowledge of AI. A majority (n=38, 80.8%) were positive/somewhat positive towards integrating AI in mammography screening. Still, many considered there to be potential risks to a high/somewhat high degree (n=16, 34.1%) or were uncertain (n=16, 34.0%). Several important uncertainties were identified, such as defining liable actor(s) when AI is integrated into medical decision-making.

**Conclusions:**

Swedish breast radiologists are largely positive towards integrating AI in mammography screening, but there are significant uncertainties that need to be addressed, especially regarding risks and responsibilities. The results stress the importance of understanding actor-specific and context-specific challenges to responsible implementation of AI in healthcare.

**WHAT IS ALREADY KNOWN ON THIS TOPIC**

- Radiologists believe that artificial intelligence (AI) will have a major impact in their field, and clinical retrospective studies of AI in mammography screening show promising results.

**WHAT THIS STUDY ADDS**

- The social, ethical and legal aspects of integrating AI in mammography screening are underexplored, and by investigating the views of breast radiologists, this study provides important insights for a responsible approach to AI in mammography screening.
- The study shows that most Swedish breast radiologists are positive about integrating AI in mammography screening, especially those with a heavy screen-reading workload. However, there is no unified vision of how AI should be used in the screening-work flow, and there is high uncertainty, and diverse views, on important aspects such as potential risks involved, and which actor(s) are liable for medical decision-making, particularly when AI is used as stand-alone reader.

**HOW THIS STUDY MIGHT AFFECT RESEARCH, PRACTICE OR POLICY**

- This study adds to the emerging body of research on AI in medical decision-making, taking into account contextual and actor-specific factors, and emphasises the social, ethical and legal unclarities of integrating AI into mammography screening, that must be addressed.

## Introduction

In radiology, the use of artificial intelligence (AI) is rapidly evolving. One area targeted as especially promising is mammography screening.^1–3^ The benefit of population-based screening is early breast cancer detection, reducing mortality and morbidity. This is a benefit balanced by the harm of false positives, overdiagnosis and false negatives.^4 5^ The vast majority of individuals who are screened do not have breast cancer, however, screen examinations are, in European guidelines, recommended to be double-read to ensure a high sensitivity.^6^ The hope is that integrating AI will result in a more efficient screening with reduced workload and a potentially higher accuracy. By adapting single-reading and double-reading to AI risk scores, or combining it with automated reading of low-risk examinations, it is suggested that the workload may be reduced by up to 63%.^7^ In theory, reducing the number of exams that are double-read will lead to fewer false positives.^8^ Retrospective studies have also shown that AI could potentially lower the false-negative rate,^9 10^ but prospective studies are still needed to understand the real impact of AI.^11^

Beyond clinical aspects, there are unresolved issues regarding the ethical, social and legal consequences of integrating AI into healthcare.^12 13^ These include how to safeguard values of medical ethics such as fairness, accountability and transparency.^14 15^ These matters are perceived as some of the greatest hurdles of implementing AI in radiology.^16–19^ In response, standards for AI in radiology are stressed, including the equal distribution of benefits and harms between stakeholders, transparency of AI-systems, curtailing bias in decision-making, and that accountability should remain with humans.^12^

The expectation that AI will change the field of radiology in the near future is highly prevalent among radiologists, trainees and medical students.^16–18^ Still, different stakeholders’ notions of the challenges are in need of more exploration.^20–22^ Prior studies show high willingness to use AI in clinical practice, but this could differ depending on subfield. While included in studies as one subdiscipline of many, not much focus has been dedicated specifically to breast radiologists, a group likely to be involved in AI-implementation on a large scale, and with experience of the particular conditions of the screening process.^16 23^ In addition, mammography screening is a major medical intervention that affects a large part of the population, and social and ethical implications of integrating AI in this context are underexplored.^19 22^ Therefore, we are examining the views of breast radiologists. Moreover, Sweden is an especially relevant case, as it is one of the most digitalised countries in the European Union,^24^ as well an early adopter of population-based breast cancer screening and with ongoing pioneering prospective trials on AI in screening.^25^

This study investigated Swedish breast radiologists’ views on the use of AI in mammography screening and their perceptions of the risks, benefits and responsibilities of actors involved, and its impact on the profession.

## Method

An online survey (using Sunet Survey) was distributed to the Swedish Society of Breast Imaging (SSBI), in which the vast majority of Swedish breast radiologists are members. The survey was conducted over the course of 1 month in the late fall of 2021. Informed consent was obtained before answering the survey, by click-response. The questionnaire contained 45 questions falling under different themes. Besides background questions used to establish respondent characteristics, questions were chosen due to their relevance for the social, ethical and legal issues of AI implementation. This included (but was not limited to): attitudes about AI-supported mammography screening, responsibility of AI-use and the future professional impact of AI integration (see online supplemental appendix A). Background questions had categorical response options. Two questions only had free-text response option. The remaining questions had Likert-scale response options, representing degrees (to a low degree, to a somewhat low degree, uncertain, to a somewhat high degree, to a high degree) or attitudes (negative, somewhat negative, uncertain, somewhat positive, positive). In addition, the respondents had the opportunity to provide free-text comments.

10.1136/bmjhci-2022-100712.supp1 Supplementary data

Results were analysed using descriptive statistics and are presented in percentages and frequencies. Correlation analyses were performed by Spearman’s r, with 95% CI and p values of <0.05 considered statistically significant. In addition, cross-tabulations were used to cover more correlations. Statistical analyses were performed using IBM SPSS Statistics for Mac (V.28.0, IBM). All free-text responses and comments were analysed using an inductive approach and used as method triangulation complementing the quantitative results. Since comments and the two free-text questions were optional, not all respondents’ views are accounted for; still, they provide valuable means for obtaining a deeper understanding.

## Results

Out of the 105 members of the SSBI, 47 answered the survey (response rate: 44.8%). Of these, 25 were females (53.2%), and the majority of the respondents were older (66.5%>50 years of age), most had long experience in breast imaging (70.2%>11 years of experience) and a high reading-volume was fairly common (38.3% performed >10 000 screen-readings per year). A majority (n*=*33, 73.3%) of the respondents reported that they sometimes, often or always had difficulties finding time to do screen-readings. More respondents estimated to have higher literacy of technology in everyday life and at work in general, than of AI. Most (n=18, 38.3%) estimated their AI literacy to be neither high nor low, and 25.5% that it was somewhat high or high. Correspondingly, 21.3% had extensive or somewhat extensive experience of using AI in their work, while nearly half (n=22, 46.8%) had no experience (table 1).

**Table 1:** Background characteristics of participating breast radiologists

Characteristics of respondents	N (%)
Age (Q1)	(N=47)
<30	0 (0)
31–40	3 (6.4)
41–50	13 (27.7)
51–60	13 (27.7)
>60 years	18 (38.3)
Gender (Q2)	(N=47)
Female	25 (53.2)
Male	22 (46.8)
Experience of breast radiology (Q3)	(N=47)
<5 years	5 (10.6)
5–10 years	9 (19.1)
11–20 years	10 (21.3)
21–30 years	11 (23.4)
>30 years	12 (25.5)
Screen readings approx. performed per year (Q4)	(N=47)
None	2 (4.3)
<2000	4 (8.5)
2000–5 000 000	5 (10.6)
5000–10 000 000	18 (38.3)
>10 000	18 (38.3)
Difficulties finding time to perform screen-readings (Q5)	(N=45)
Never	4 (8.9)
Seldom	8 (17.8)
Sometimes	19 (42.2)
Often	8 (17.8)
Always	6 (13.3)
Self-estimated technology literacy, everyday life (Q6)	(N=47)
Low	0 (0)
Somewhat low	1 (2.1)
Neither high nor low	20 (42.6)
Somewhat high	19 (40.4)
High	7 (14.9)
Self-estimated technology literacy, work (Q7)	(N=47)
Low	0 (0)
Somewhat low	1 (2.1)
Neither high nor low	17 (36.2)
Somewhat high	21 (44.7)
High	8 (17.0)
Self-estimated AI literacy (Q8)	(N=47)
Low	4 (8.5)
Somewhat low	13 (27.7)
Neither high nor low	18 (38.3)
Somewhat high	8 (17.0)
High	4 (8.5)
Experience of using AI in breast radiology (Q12)	(N=47)
None	22 (46.8)
Little	9 (19.1)
Somewhat little	6 (12.8)
Somewhat large	6 (12.8)
Large	4 (8.5)

AI, artificial intelligence.

### Attitudes, benefits and risks of AI in mammography screening

#### Positive views and potential benefits

**Figure 1:**
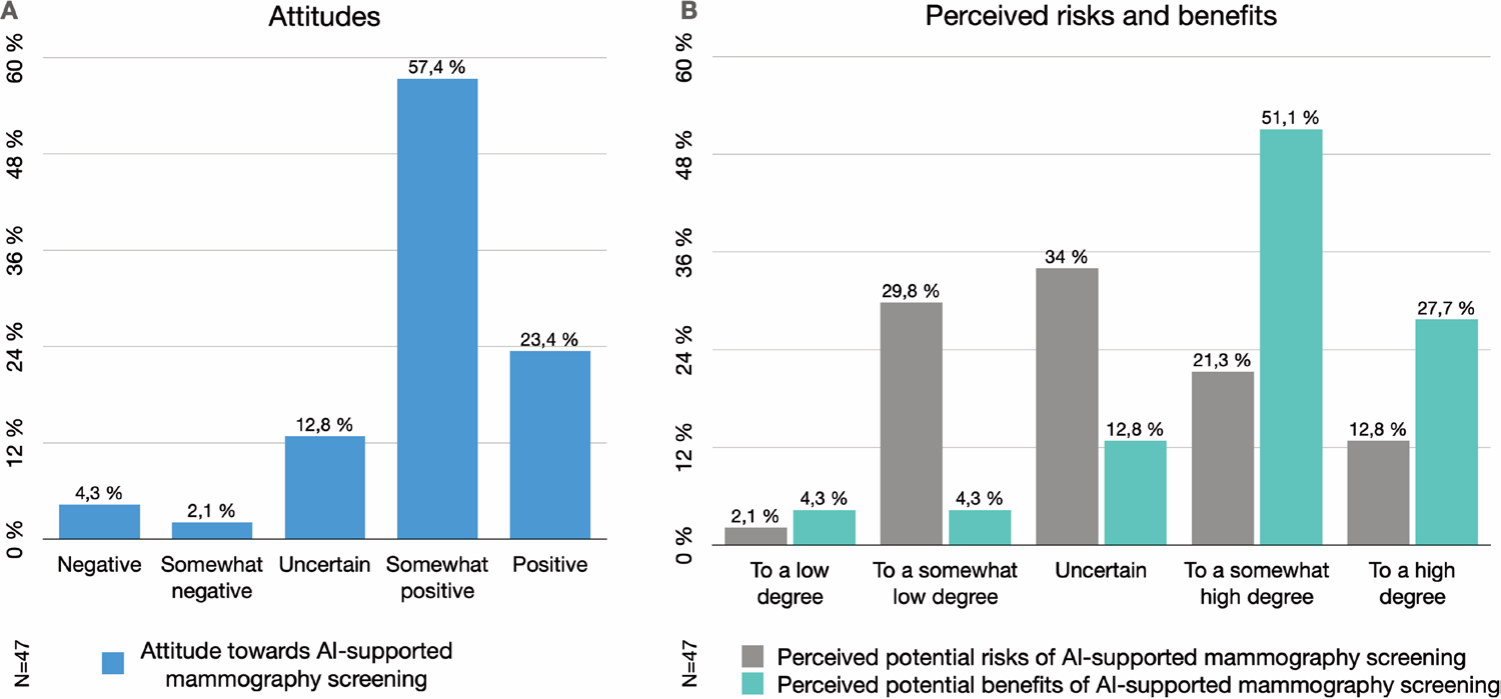
Attitudes. (A) The Swedish breast radiologists’ attitude towards AI-supported mammography screening (Q9). (B) The perceived degree of benefits and risks of AI-supported mammography screening (Q10 and Q11). AI, artificial intelligence.

**Figure 2:**
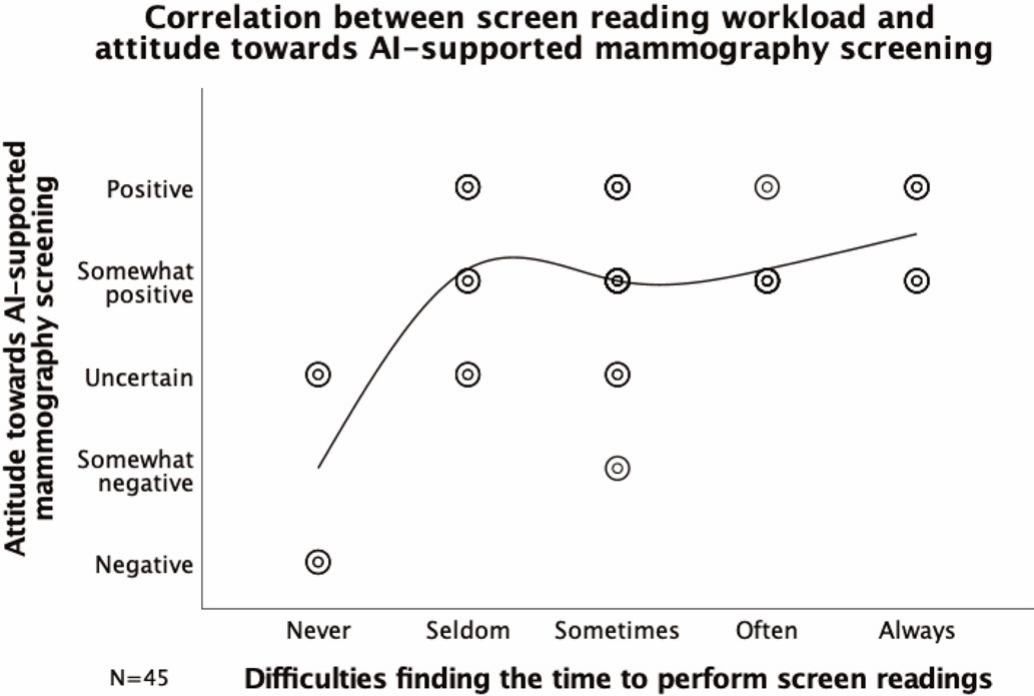
Correlation between the Swedish breast radiologists’ attitude towards AI supported mammography screening (Q9) and their difficulties to find the time to perform screen-readings (Q5) (Spearman’s r=0.367, 95% CI, p=0.013). The results suggest that the Swedish breast radiologists’ experienced screen-reading workload correlates with their attitude towards AI-supported mammography screening. AI, artificial intelligence.

The breast radiologists were, to a large extent, positive towards AI-supported mammography screening; 80.8% (n=38) being somewhat positive or positive (table 2, figure 1A). Comments suggest that AI is perceived as a good complement and solution to the scarcity of breast radiologists. Furthermore, having difficulties finding the time to perform screen-readings correlated with a positive attitude towards AI-supported screening (Spearman’s r=0.367, 95% CI, p=0.013, figure 2). A correlation between self-estimated literacy of AI and attitude could not be established (p=0.825).

**Table 2:** General attitudes and perceived potential benefits and risks of AI-supported mammography screening

Attitudes, perceived benefits and risks	N (%)
Attitude towards AI-supported mammography screening (Q9)	(N=47)
Positive	11 (23.4)
Somewhat positive	27 (57.4)
Uncertain	6 (12.8)
Somewhat negative	1 (2.1)
Negative	2 (4.3)
Preferred use of AI in mammography screening (Q13)	(N=47)
AI as triage tool	6 (12.8)
AI as stand-alone reader	2 (4.3)
AI as replacement of one in double reading	21 (44.7)
AI as addition to double reading	14 (29.8)
Not at all	4 (8.5)
Potential benefits of AI-supported screening (Q10)	(N=47)
To a high degree	13 (27.7)
To a somewhat high degree	24 (51.1)
Uncertain	6 (12.8)
To a somewhat low degree	2 (4.3)
To a low degree	2 (4.3)
Potential risks of AI-supported screening (Q11)	(N=47)
To a high degree	6 (12.8)
To a somewhat high degree	10 (21.3)
Uncertain	16 (34.0)
To a somewhat low degree	14 (29.8)
To a low degree	1 (2.1)
Perceived risk of overconfidence in AI assessments (Q15)	(N=47)
To a high degree	4 (8.5)
To a somewhat high degree	9 (19.1)
Uncertain	20 (42.6)
To a somewhat low degree	13 (27.7)
To a low degree	1 (2.1)
Perceived risk of non-representative training data (Q38)	(N=47)
To a high degree	2 (4.3)
To a somewhat high degree	9 (19.1)
Uncertain	24 (51.1)
To a somewhat low degree	9 (19.1)
To a low degree	3 (6.4)
Perceived risk of inferior AI performance on certain risk groups or specific type of cases (Q39)	(N=47)
To a high degree	6 (12.8)
To a somewhat high degree	13 (27.7)
Uncertain	22 (46.8)
To a somewhat low degree	5 (10.6)
To a low degree	1 (2.1)

AI, artificial intelligence.

A majority (n=37, 78.7%) of the respondents believed that there were potential benefits in using AI-supported screening, to a somewhat high or high degree (figure 1B). Benefits specified in the comments were improved detection and consistency in screen-reading. The respondents favoured using AI as a replacement of 1 reader in double-reading (n=21, 44.7%) or in addition to 2 human readers (n=14, 29.8%) (table 2). A wish to combine triage, reader replacement and detection support were also mentioned in the comments.

#### Negative views and potential risks

Nearly one-fifth of respondents (n*=*9, 19.2%) were negative/somewhat negative or uncertain about AI-supported screening (table 2). In the comments, negative attitudes refer to experiencing AI as linked to large numbers of false positives (due to a high sensitivity for calcifications), difficulty in interpreting AI-assessments and the risk of an increased workload, as expressed by one respondent:

*It was annoying to have to go back and assess different AI findings of benign things all the time […] that I would normally not have had to put any energy into assessing. It made the work slower and disrupted the work pace, leading to more exhaustion* [P12].

The views concerning potential risks of integrating AI were diverse (figure 1B). Comments revealed that, besides medical risks, some feared AI would lead to a deterioration of working conditions, an increase in false positives and interpretation load, and loss of competence due to a lack of continuous training on healthy mammograms:

*Consultation hours with ultrasounds and biopsies are often heavily booked with worried patients. Working whole days like that would be hard*. [P12]

*There is a risk that AI detects findings that are obviously benign, which will take time and effort to investigate and prove. Some changes that are unquestionable to a radiologist, AI can’t see* [P23].

Other comments stressed legal and ethical risks. One case mentioned, was if a physician disregards an AI finding that later turns out to be a cancer. The respondent suggested radiologists will be put in that situation ‘all the time’ since AI detects so many findings.

About half of the respondents (n=24, 51.1%) were uncertain as to whether there are risks in AI-models being trained on data that are not representative of the population to which they are applied. Many were also uncertain as to whether AI-models perform poorly on risk groups or certain types of cases (n=22, 46.8%) (table 2). Cases perceived as possibly more difficult for AI to assess included; dense breasts, atypical soft tissue masses without calcification, architectural distortion, developing asymmetric density, postoperative changes or young individuals with hereditary risk.

### Accountability of AI-use

**Figure 3:**
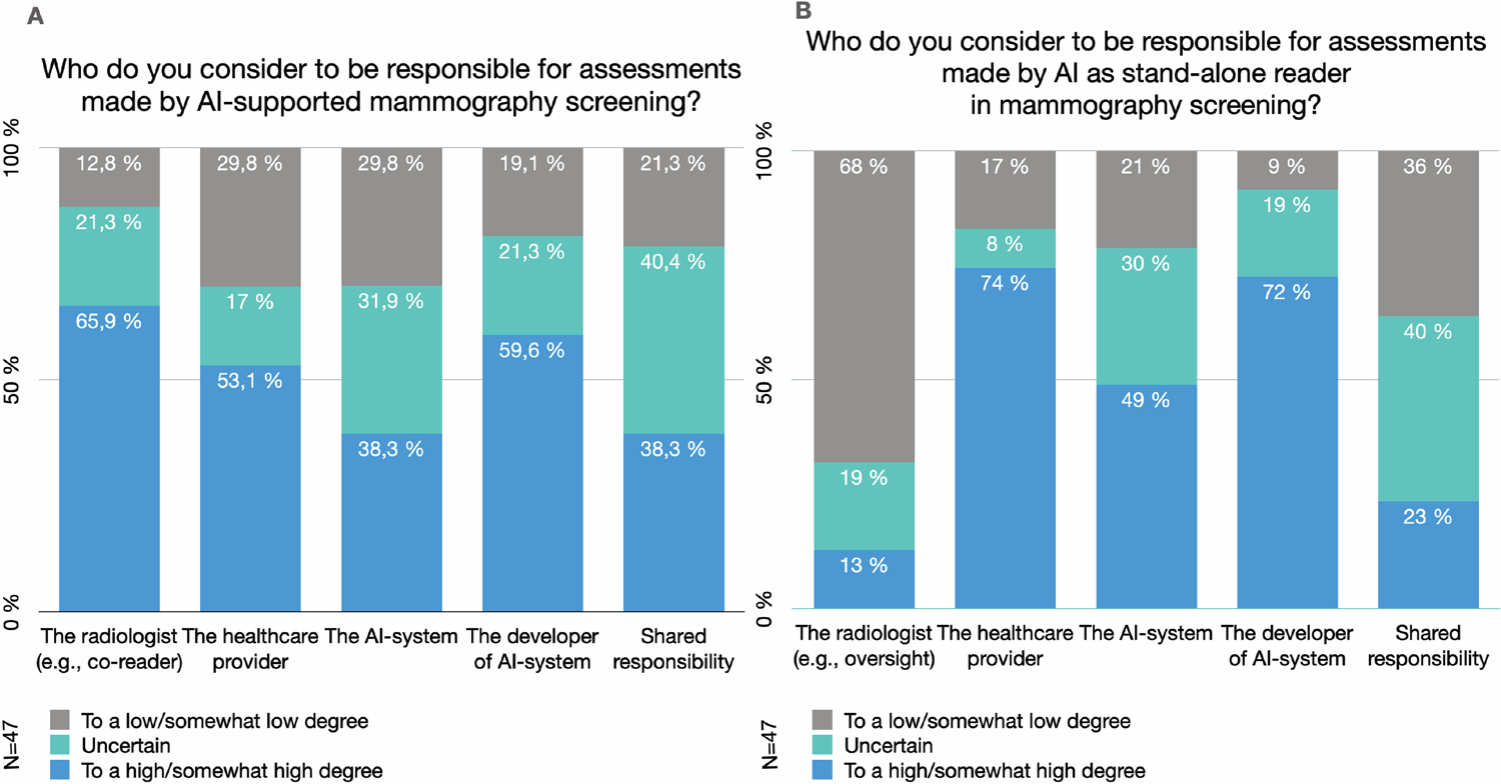
Accountability. (A) The Swedish breast radiologists’ perceived levels of accountability of different actors for assessments made by AI-supported mammography screening (Q17–Q21). (B) The Swedish breast radiologists’ perceived levels of accountability of different actors for assessments made by AI as stand-alone reader in mammography screening (Q22–Q26). AI, artificial intelligence.

When AI is used in addition to radiologists in screen reading, most the respondents (n=31, 65.9%) considered the radiologist to be responsible for the assessments to a high/somewhat high degree, but 21.3% (n=10) were uncertain (figure 3A). When AI is used as a stand-alone reader, the radiologist (eg, in terms of oversight) was considered responsible to a high/somewhat high degree only by 12.8% (n=6) (figure 3B). The healthcare provider was, to a larger extent, considered responsible when AI is stand-alone reader, compared with when it is used in addition to radiologist(s). This was also the case regarding the responsibility of developers of AI-systems (figure 3).

To answer whether agency was ascribed to the AI-system, as is common in everyday discussions about AI, we included it as an option among liable actors. When used in addition to radiologist(s), 38.3% (n=18) of the respondents considered the AI-system to be responsible to a high/somewhat high degree. When used as stand-alone reader, the number was larger (n=23, 48.9%) and about one-third of the respondents were uncertain (n=14, 29.8%) (figure 3). Perceived shared responsibility was less prevalent when AI is used as a stand-alone reader. Uncertainty and urgency on the issue of responsibility emerged in the comments: *This is the most difficult part. Who takes responsibility? Healthcare should do it, probably, but it is actually the AI-system and the AI-developer who should be accountable for the result* [P24].

### Impact on the profession

Nearly half of the breast radiologists in the sample (n=21, 44.7%) believed that integrating AI in mammography screening would encompass substantial differences in comparison to other previously introduced technologies (such as digital mammography and tomosynthesis), to a high/somewhat high degree. A comment suggested the reason for this was that previous technologies aimed to improve image quality, while AI is about delegating assessments to the technology. However, more than one-third of the respondents (n=17, 36.2%) were uncertain as to whether there were any substantial differences of introducing AI.

Moreover, there was a mix of viewpoints regarding how integrating AI might impact the role of the breast radiologist. Most commonly (n=20, 42.6%) AI was believed to have no impact, while nearly a third of respondents (n=15, 31.9%) thought it would strengthen/somewhat strengthen the role of breast radiologists and 25.5% (n=12) that it would weaken/somewhat weaken it. Only 21.3% (n=10) believed the use of AI would make it easier to recruit new breast radiologists to a high/somewhat high degree.

#### Relation to screening participants

The question about whether implementing AI-supported mammography screening would impact the relationship with screening participants was answered using free-text responses. Out of the total sample, 32 respondents answered, and both positive and negative outlooks were articulated. Some stated that the use of AI would increase the participants’ trust, and improve working conditions and thereby also the relationship with caretakers. Other respondents suggested that trust would decrease and introducing AI would ‘lead to chaos’ and ‘waste everyone’s time’. Several highlighted the importance of AI systems being valid and trustworthy, and to be able to convey that trustworthiness to relevant actors. Some respondents also emphasised the significance of having radiologists in charge of AI implementation, management and quality control.

#### Technological development

How the profession will evolve, in light of current technological development, provided a mix of viewpoints. Some pointed to socioeconomic factors, such as: *[I] think that AI will be implemented in screening considering the economic benefits it could have for the employers* [P33]. Several respondents voiced insecurities and expressed reservations:

*It probably cannot be avoided in the long run and should be able to provide more time for what needs to be investigated or acted upon. I am a bit worried about the loss of knowledge of the “normal breast as background”* [P2].

Still, others emphasised that they considered AI as not yet reaching an acceptable performance level: *until AI becomes good enough, it will be a long way* [P14]. However, other responses expressed hopes of what AI-integration could bring; easing screen-reading workload, improving diagnostics and healthcare quality, with statements like: *AI will be able to sort out the easier cases, decrease workload, and help to find more cancers* [P36]. Moreover, some comments emphasised AI’s supporting qualities:

*It feels good to be able to be supported by AI in the screening, it could be nice to have a second reader (AI) whom never loses concentration. Considering the screening volumes and the scarcity of breast radiologists it feels good that AI can complement us*. [P16]

## Discussion

In this study, we have investigated Swedish breast radiologists’ views on the use of AI in mammography screening. The respondents were, to a large extent, positive towards the integration of AI in screen reading, especially those having difficulties finding the time to perform screen-reading. This could explain the slightly more positive attitude, compared with general studies on radiologists’ attitudes towards AI.^23 26 27^ We could not establish a correlation between attitude and AI-literacy, prevalent in previous general studies.^23^ However, it needs to be taken into account that our sample represents a relatively small number of individuals. Those more opinionated about AI could also be more inclined to answer the survey, possibly inducing bias in the results. The specific context, of mammography screening and the profession of breast radiologists, in a digitally advanced welfare state, however, showcases the importance of considering technological implementations in relation to organisational and socioeconomic structures.

Furthermore, we did identify important reservations, factors associated with high uncertainties, and diverse viewpoints, such as regarding liability of AI use. The question is whether established practices need to be adjusted when medical decisions are increasingly supported by automated technologies or AI-systems. Our results point to a somewhat higher perceived responsibility of radiologists in AI-supported radiological practice, compared with previous studies.^20 27^ Furthermore, the results show the complexity of accountability when AI enters radiology, how it is contextual, dependent on how AI is used and which actors are included. This further emerges in the insecurities regarding liability for missed cancers, when AI is used as a co-reader or as stand-alone reader, or when radiologists disregard AI findings. The results indicate a perceived shift of responsibility away from the radiologist as automation increases. Additionally, uncertainties regarding the responsibilities of AI-developers (and AI-systems) suggest a need for clarification.^28^

We could not identify one unified vision of a preferred way to use AI in mammography screening. Previously, AI has been expected to be used as second reader and for optimising workflows.^16–18^ While using AI as replacement for one reader in double-reading was the most preferred option in our study, many favoured using it as an addition to double-reading or in a combination of uses, suggesting perceived qualities other than workload reduction. Furthermore, the perceived risk of AI deteriorating working conditions might be due to several reasons. Besides a risk of eroded knowledge of the normal breast, reduced screen reading workload might not improve working conditions. While more time for patient-centred care is portrayed as a positive outcome of AI,^27^ some perceived screen reading as a welcomed interruption from emotionally burdensome work, which might be lost due to automation. Working with AI-systems also adds layers of interpretation,^29^ which could be exhausting. This seems to be perceived as a medical risk, but also as an ethical burden with legal uncertainties.

Additionally, AI in mammography screening needs to be considered in light of previous innovations. Some aspects are not unique for AI, such as contested expertise.^30^ However, radiologists, trainees and medical students strongly expect AI to change the field and impact job opportunities, tasks and relationships with patients.^16–18 20^ Our study shows that breast radiologists believed that AI will impact the profession, both positively and negatively. However, most did not believe it would impact the role of the breast radiologist. Few thought it would improve recruitment, possibly due to the idea of AI negatively affecting the professional reputation.^26^ Many considered, or were uncertain whether, implementing AI represents a substantial difference in comparison to previous technologies. While new imaging methods aim to improve cancer visibility, AI differs since it involves medical decision-making. This implies that social, ethical and legal aspects have to be addressed, which in turn depends on how AI is incorporated into the clinical workflow. Greater unclarity about accountability seems to be prevalent regarding AI as a stand-alone technique, which was also the least favoured approach. This suggests that physicians are not willing to renounce their responsibility in medical decision-making. In total, our results echo the need for more research on social, ethical and legal matters of integrating AI into radiology and screening.

### Strengths and limitations

The main limitations of the study are the specific conditions of the Swedish setting and the small number of respondents. The response rate was satisfactory, but the target population was limited since there are few Swedish breast radiologists. The study’s strengths were that the respondents were highly experienced in breast imaging and that half of the group had experience of using AI in breast imaging.

## Conclusions

Breast radiologists in Sweden were largely positive about integrating AI in mammography screening, especially those with a heavy screen-reading workload, citing reduced workload and increased sensitivity as benefits. Still, we identified several concerns and uncertainties that need to be addressed, foremost regarding potential risks – pertaining to medical outcomes, working conditions and the question of liability in medical decision-making when using AI. Furthermore, there is a lack of consensus on the optimal use of AI in the screening workflow. The results emphasise the need to understand actor-specific and context-specific challenges for responsible implementation of AI.

## Data Availability

No data are available. Data cannot be openly shared, due to respondent anonymity.
