# Supplementary material for: Anticipating artificial intelligence in mammography screening: views of Swedish breast radiologists
Source: BMJ Health Care Inform. 2023 May 22;30(1):e100712. doi: 10.1136/bmjhci-2022-100712 (PMC10230899; doi:10.1136/bmjhci-2022-100712)
Supplement: Supplementary data [file bmjhci-2022-100712supp001.pdf]

Enkät: Bröstradiologers syn på användning av artificiell intelligens i mammografiscreening

1. Ålder

- Under 31 år
- 31-40 år
- 41-50 år
- 51-60 år
- Över 60 år

2. Kön

- Kvinna
- Man
- Annat

3. Hur lång erfarenhet har du av bröstradiologi?

- Under 5 år
- 5-10 år
- 11-20 år
- 21-30 år
- Över 30 år

Kommentar

4. Hur många mammografiscreeningundersökningar granskar du uppskattningsvis per år?

- Inga
- Färre än 2 000
- 2 000 - 5 000
- 5 000 - 10 000
- Fler än 10 000

Kommentar

5. Upplever du att det är svårt att hinna granska screeningundersökningarna?

- Aldrig
- Sällan
- Ibland
- Ofta
- Alltid

Kommentar

6. Hur bedömer du din egen teknikkunnighet generellt i vardagen?

- Låg
- Ganska låg
- Varken hög eller låg
- Ganska hög
- Hög

Kommentar

7. Hur bedömer du din egen teknikkunnighet generellt i arbetet?

- Låg
- Ganska låg
- Varken hög eller låg
- Ganska hög
- Hög

Kommentar

8. Hur bedömer du din egen kunskap om AI, artificiell intelligens?

- Låg
- Ganska låg
- Varken hög eller låg
- Ganska hög
- Hög

Kommentar

Inställning till artificiell intelligens i arbetet

9. Vilken är din inställning till användning av AI-stödd mammografiscreening?

- Negativ
- Ganska negativ
- Tveksam
- Ganska positiv
- Positiv

Kommentar

10. Anser du att det finns potentiella vinster med AI-stödd mammografiscreening?

- I låg grad
- I ganska låg grad
- Tveksam
- I ganska hög grad
- I hög grad

Kommentar

11. Anser du att det finns potentiella risker med AI-stödd mammografiscreening?

- I låg grad
- I ganska låg grad
- Tveksam
- I ganska hög grad
- I hög grad

Kommentar

12. Vilken erfarenhet har du av att använda AI i ditt arbete som mammografiläkare?

- Ingen
- Liten
- Ganska liten
- Ganska stor
- Stor

Kommentar

13. Hur skulle du vilja använda AI i mammografiscreening?

- AI som verktyg för triage
- AI som enda granskare
- AI ersätter en granskare vid dubbelgranskning
- AI används i tillägg till dubbelgranskning
- Inte alls

Det finns flera olika möjligheter att använda AI i screeningen. Skriv gärna egna förslag eller kommentarer till ovan nämnda förslag, t.ex. hur du skulle vilja kombinera alternativen.

14. I vilken grad skulle du lita på AI-verktygs bedömningar?

- I låg grad
- I ganska låg grad
- Tveksam
- I ganska hög grad
- I hög grad

Kommentar

15. Tror du att det finns en risk att radiologer har en övertro till AI-verktygs bedömningar?

- I låg grad
- I ganska låg grad
- Tveksam
- I ganska hög grad
- I hög grad

Kommentar

16. Skulle din tillit till AI-verktyget vara större ifall radiologernas bedömningar bidrog till fortsatt träning av AI-verktyget efter klinisk implementering?

- I låg grad
- I ganska låg grad
- Tveksam
- I ganska hög grad
- I hög grad

Kommentar

Vem anser du bär ansvaret för bedömningar gjorda vid AI-stödd mammografiscreening?

17. Den enskilda radiologen (ex. i egenskap av medgranskare)

- I låg grad
- I ganska låg grad
- Tveksam
- I ganska hög grad
- I hög grad

18. Vårdgivaren

- I låg grad
- I ganska låg grad
- Tveksam
- I ganska hög grad
- I hög grad

19. AI-verktyget

- I låg grad
- I ganska låg grad
- Tveksam
- I ganska hög grad
- I hög grad

20. Utvecklare av AI-verktyget

- I låg grad
- I ganska låg grad
- Tveksam
- I ganska hög grad
- I hög grad

21. Delat ansvar

- I låg grad
- I ganska låg grad
- Tveksam
- I ganska hög grad
- I hög grad

Om du anser att det är delat ansvar, specificera mellan vilka aktörer och varför:

Vem anser du bär ansvar för bedömningar gjorda vid mammografiscreening med enbart AI-granskning?

22. Den enskilda radiologen (ex. i egenskap av översyn av screeningprocessen)

- I låg grad
- I ganska låg grad
- Tveksam
- I ganska hög grad
- I hög grad

23. Vårdgivaren

- I låg grad
- I ganska låg grad
- Tveksam
- I ganska hög grad
- I hög grad

24. AI-verktyget

- I låg grad
- I ganska låg grad
- Tveksam
- I ganska hög grad
- I hög grad

25. Utvecklare av AI-verktyget

- I låg grad
- I ganska låg grad
- Tveksam
- I ganska hög grad
- I hög grad

26. Delat ansvar

I låg grad I ganska låg grad Tveksam I ganska hög grad

I hög grad

Om du anser att det är delat ansvar, specificera mellan vilka aktörer och varför:

Om du använder/har använt AI i ditt arbete, anser du att du har fått den information du behöver för att kunna bedöma riktigheten i AI-verktygets bedömning:

27. Vid användning av verktyget (information i användargränssnitt m.m.)?

I låg grad I ganska låg grad Tveksam I ganska hög grad

I hög grad

Kommentar

28. Inför användning (information från utvecklare m.m.)?

I låg grad I ganska låg grad Tveksam I ganska hög grad

I hög grad

Kommentar

29. Om du skulle använda AI-verktyg i mammografiscreening, önskar du få information om hur det har utvecklats (exempelvis vilken data som verktyget har tränats på)?

- I låg grad
- I ganska låg grad
- Tveksam
- I ganska hög grad
- I hög grad

Kommentar

Tror du att följande information hade underlättat för dig att kunna avgöra tilliten till ett AI-verktygs bedömning:

30. Information om vad i bilden som föranlett en viss riskgradering?

- I låg grad
- I ganska låg grad
- Tveksam
- I ganska hög grad
- I hög grad

Kommentar

31. Information om vad och hur mycket i bilden som hade behövt vara annorlunda för att den istället skulle ha graderats som en högre respektive lägre risknivå?

- I låg grad
- I ganska låg grad
- Tveksam
- I ganska hög grad
- I hög grad

Kommentar

32. Information om bakomliggande kod/algoritmer?

- I låg grad
- I ganska låg grad
- Tveksam
- I ganska hög grad
- I hög grad

Kommentar

33. Information om vilka kompetenser (ex. bröstradiolog, datavetare, statistiker) som varit delaktiga i utvecklingen av AI-verktyget?

- I låg grad
- I ganska låg grad
- Tveksam
- I ganska hög grad
- I hög grad

Kommentar

34. Information om vilken data AI-modellen har tränats på?

- I låg grad
- I ganska låg grad
- Tveksam
- I ganska hög grad
- I hög grad

Kommentar

35. Information om hur eventuell uppmärkning av träningsdata gått till (t.ex. hur mammografier med cancer har identifierats och annoterats inför träning av AI)?

- I låg grad
- I ganska låg grad
- Tveksam
- I ganska hög grad
- I hög grad

Kommentar

36. Information om verktygets fortsatta lärande efter klinisk implementering?

- I låg grad
- I ganska låg grad
- Tveksam
- I ganska hög grad
- I hög grad

Kommentar

37. Tror du att du skulle ha kompetens att avgöra om ett AI-verktygs bedömning vid mammografiscreening är korrekt?

- I låg grad
- I ganska låg grad
- Tveksam
- I ganska hög grad
- I hög grad

Kommentar

38. Tror du att det finns risk att de data som AI-verktyg har tränats på vid utveckling inte är representativ för den befolkningsgrupp som verktyget appliceras på?

- I låg grad
- I ganska låg grad
- Tveksam
- I ganska hög grad
- I hög grad

Kommentar

39. Tror du att det finns en risk att AI-verktyg presterar sämre än radiologer vid mammografiscreening för vissa riskgrupper eller specifika typer av fall?

- I låg grad
- I ganska låg grad
- Tveksam
- I ganska hög grad
- I hög grad

Beskriv i så fall vilka typer av riskgrupper/fall:

Professionens utveckling

40. Om du skulle använda AI-verktyg i ditt arbete med mammografiscreening, skulle du önska att din utvärdering av verktygets bedömning skulle användas för att fortsätta träna och förbättra verktygets precision?

- I låg grad
- I ganska låg grad
- Tveksam
- I ganska hög grad
- I hög grad

Kommentar

41. Anser du att det finns betydande skillnader i att implementera AI-verktyg i mammografiscreening jämfört med annan tidigare teknikutveckling (t.ex. digital mammografi, tomosyntes)?

- I låg grad
- I ganska låg grad
- Tveksam
- I ganska hög grad
- I hög grad

Kommentar

42. Hur tror du att mammografiläkarens roll skulle förändras om AI-stödd mammografiscreening införs?

- Försvagas
- Försvagas något
- Inte alls
- Stärkas något
- Stärkas

Kommentar

43. Tror du att införande av AI-stödd mammografiscreening skulle göra det lättare att rekrytera nya bröstradiologer?

- I låg grad
- I ganska låg grad
- Tveksam
- I ganska hög grad
- I hög grad

Kommentar

44. Tror du att införande av AI-stödd mammografiscreening skulle påverka relationen mellan läkare/sjukvård och individer som deltar i screening? I så fall, hur?

45. Hur tror du att professionen kommer att utvecklas, med tanke på rådande teknikutveckling med AI?

Survey: Breast Radiologists' Views on the use of Artificial Intelligence in Mammography Screening

1. Age

- <31 years
- 31-40 years
- 41-50 years
- 51-60 years
- >60 years

2. Gender

- Femal
- Male
- Other

3. How long experience do you have of working with breast radiology?

- <5 years
- 5-10 years
- 11-20 years
- 21-30 years
- >30 years

Comments

4. How many screening exams do you approximately review per year?

- None
- <2000
- 2 000 - 5 000
- 5 000 - 10 000
- >10 000

Comments

5. Do you experience difficulties finding the time to perform the screen-reading?

- Never
- Seldom
- Some-times
- Often
- Always

Comments

6. What is your estimated level of literacy of technology in everyday life?

- Low
- Somewhat low
- Neither high nor low
- Somewhat high
- High

Comments

7. What is your estimated general level of literacy of technology at work?

- Low
- Somewhat low
- Neither high nor low
- Somewhat high
- High

Comments

8. What is you estimated level of literacy of AI, artificial intelligence?

- Low
- Somewhat low
- Neither high nor low
- Somewhat high
- High

Comments

Attitudes towards artificial intelligence, AI, in the work

9. What is your attitude towards the use of AI-supported mammography screening?

- Negative
- Somewhat negative
- Uncertain
- Somewhat positive
- Positive

Comments

10. Do you consider that there are potential benefits of using AI-supported mammography screening?

- To low degree
- To somewhat low degree
- Uncertain
- To somewhat high degree
- To high degree

Comments

11. Do you consider that there are potential risks of using AI-supported mammography screening?

- To low degree
- To somewhat low degree
- Uncertain
- To somewhat high degree
- To high degree

Comments

12. How much experience do you have of using AI in breast radiology?

- ○ None
- ○ Little
- ○ Somewhat little
- ○ Somewhat large
- ○ Large

Comments

13. How would you like to use AI in mammography screening?

- ○ AI as triage tool
- ○ AI as stand-alone reader
- ○ AI as replacement of one radiologist in double screen-reading
- ○ AI as addition to double screen-reading
- ○ Not at all

There are several possibilities of how to use AI in screening, please, write your suggestion or comments to the options mentioned above, for example how you would like to combine different options.

14. To what degree would you trust the assessments made by an AI-system?

- ○ To low degree
- ○ To somewhat low degree
- ○ Uncertain
- ○ To somewhat high degree
- ○ To high degree

Comments

15. Do you think there is a risk of radiologists over-relying on assessments made by AI-systems?

- ○ To low degree
- ○ To somewhat low degree
- ○ Uncertain
- ○ To somewhat high degree
- ○ To high degree

Comments

16. Would your trust in the AI-system be increased if your assessment contributed to train the AI-system after clinical implementation?

- To low degree
- To somewhat low degree
- Uncertain
- To somewhat high degree
- To high degree

Comments

Whom do you consider being responsible for assessments made by AI-supported mammography screening?

17. The radiologist (e.g., as co-reader)

- To low degree
- To somewhat low degree
- Uncertain
- To somewhat high degree
- To high degree

18. The health care provider

- To low degree
- To somewhat low degree
- Uncertain
- To somewhat high degree
- To high degree

19. The AI-system

- To low degree
- To somewhat low degree
- Uncertain
- To somewhat high degree
- To high degree

20. Developers of the AI-system

- To low degree
- To somewhat low degree
- Uncertain
- To somewhat high degree
- To high degree

21. Shared responsibility

- To low degree
- To somewhat low degree
- Uncertain
- To somewhat high degree
- To high degree

If you consider there to be shared responsibility, specify between which actors and why:

Whom do you consider being responsible for assessments made by AI-supported mammography screening with AI as single reader?

22. The radiologist (e.g., in terms of oversight)

- To low degree
- To somewhat low degree
- Uncertain
- To somewhat high degree
- To high degree

23. The health care provider

- To low degree
- To somewhat low degree
- Uncertain
- To somewhat high degree
- To high degree

24. The AI-tool

- To low degree
- To somewhat low degree
- Uncertain
- To somewhat high degree
- To high degree

25. The developer of the AI-system

- To low degree
- To somewhat low degree
- Uncertain
- To somewhat high degree
- To high degree

26. Shared responsibility

- To low degree
- To somewhat low degree
- Uncertain
- To somewhat high degree
- To high degree

If you consider there to be shared responsibility, specify between which actors and why:

If you are using/have been using AI in your work, do you consider that you have recieved the information you need, to be able to evaluate the accuracy of the AI-system's assessment:

27. When using the system (information in user interface etc.)?

- To low degree
- To somewhat low degree
- Uncertain
- To somewhat high degree
- To high degree

Comments

28. Prior to use of AI-system (information from developer etc.):

- To low degree
- To somewhat low degree
- Uncertain
- To somewhat high degree
- To high degree

Comments

29. If you were to use an AI system in mammography screening, would you wish to receive information about how it was developed (for example, regarding what data were used for training)?

- To low degree
- To somewhat low degree
- Uncertain
- To somewhat high degree
- To high degree

Comments

Do you think that the following information would have supported your trust evaluation of the assessments made by an AI-system:

30. Information about what in the image that caused the given risk score?

- To low degree
- To somewhat low degree
- Uncertain
- To somewhat high degree
- To high degree

Comments

31. Information about what, and how much, in the image that would have had to be different for it to receive a lower versus higher risk score?

- To low degree
- To somewhat low degree
- Uncertain
- To somewhat high degree
- To high degree

Comments

32. Information about the underlying code/algorithms?

- To low degree
- To somewhat low degree
- Uncertain
- To somewhat high degree
- To high degree

Comments

33. Information about competences involved in the development of the AI-system (e.g., breast radiologist, computer scientist, statisticians)?

- To low degree
- To somewhat low degree
- Uncertain
- To somewhat high degree
- To high degree

Comments

34. Information about training data?

- To low degree
- To somewhat low degree
- Uncertain
- To somewhat high degree
- To high degree

Comments

35. Information the labelling of the training data (e.g., how exams with cancer have been identified and annotated for the training of the AI-system)?

- To low degree
- To somewhat low degree
- Uncertain
- To somewhat high degree
- To high degree

Comments

36. Information about continuous training of the AI-system after clinical implementation?

- To low degree
- To somewhat low degree
- Uncertain
- To somewhat high degree
- To high degree

Comments

37. Do you consider that you would have competency enough to evaluate the accuracy of an AI-system's assessment of screening exams?

- To low degree
- To somewhat low degree
- Uncertain
- To somewhat high degree
- To high degree

Comments

38. Do you think there is a risk of the training data for AI-system not being representative enough for the demographics of the target population?

- To low degree
- To somewhat low degree
- Uncertain
- To somewhat high degree
- To high degree

Comments

39. Do you think there is a risk that the performance of AI-systems is lower than radiologists' regarding exams of certain risk groups or cases?

- To low degree
- To somewhat low degree
- Uncertain
- To somewhat high degree
- To high degree

If so, describe which risk groups/cases:

Development of the profession

40. If you would be using AI in your work with mammography screening, would you wish for your evaluation of the AI assessment to be included in the continuous training of the system for improving its precision?

- To low degree
- To somewhat low degree
- Uncertain
- To somewhat high degree
- To high degree

Comments

41. Do you consider there to be significant differences in implementing AI in mammography screening, in comparison to previous technologies (e.g., digital mammography, tomosynthesis)?

- To low degree
- To somewhat low degree
- Uncertain
- To somewhat high degree
- To high degree

Comments

42. How do you think the role of the breast radiologist would change if AI-supported mammography screening was implemented?

- Weakened
- Somewhat weakened
- Not at all
- Somewhat strengthened
- Strengthened

Comments

43. Do you think implementing AI-supported mammography screening would make recruitment of breast radiologists easier?

- To low degree
- To somewhat low degree
- Uncertain
- To somewhat high degree
- To high degree

Comments

44. Do you think that implementing AI-supported mammography screening would impact the relation between doctors/healthcare and screening participants? If so, how?

45. How do you think the profession will evolve, with regards to current technological development of AI?
